# Molecular mechanism of bovine Gasdermin D-mediated pyroptosis

**DOI:** 10.1186/s13567-024-01282-1

**Published:** 2024-02-27

**Authors:** Zhendong Ge, Jinxia Xu, Ke Yang, Longjian Wu, Shan Chen, Biao Chen, Jiangyao Tian, Jinpeng Zhang, Ahui Xu, Bei Huang, Houhui Song, Yang Yang

**Affiliations:** Key Laboratory of Applied Technology On Green-Eco-Healthy Animal Husbandry of Zhejiang Province, Zhejiang Provincial Engineering Research Center for Animal Health Diagnostics & Advanced Technology, Zhejiang International Science and Technology Cooperation Base for Veterinary Medicine and Health Management, China-Australia Joint Laboratory for Animal Health Big Data Analytics, College of Animal Science and Technology & College of Veterinary Medicine of Zhejiang A&F University, 666 Wusu Street, Lin’an District, Hangzhou, 311300 Zhejiang Province China

**Keywords:** Pyroptosis, Bovine Gasdermin D, inflammatory caspase

## Abstract

Pyroptosis is a form of programmed cell death characterized by cell swelling, pore formation in the plasma membrane, lysis, and releases of cytoplasmic contents. To date, the molecular mechanism of human and murine Gasdermin D-mediated pyroptosis have been fully investigated. However, studies focusing on molecular mechanism of bovine Gasdermin D (bGSDMD)-mediated pyroptosis and its function against pathogenic infection were unclear. In the present study, we demonstrate that bovine caspase-1 (bCaspase-1) cleaves bGSDMD at amino acid residue D277 to produce an N-terminal fragment (bGSDMD-p30) which leads to pyroptosis. The amino acid residues T238 and F239 are critical for bGSDMD-p30-mediated pyroptosis. The loop aa 278-299, L293 and A380 are the key sites for autoinhibitory structure of the full length of bGSDMD. In addition, bCaspase-3 also cleaves bGSDMD at residue Asp86 without inducing cell death. Therefore, our study provides the first detailed elucidation of the mechanism of bovine GSDMD-mediated pyroptosis. The results will establish a significant foundation for future research on the role of pyroptosis in bovine infectious diseases.

**Supplementary Information:**

The online version contains supplementary material available at 10.1186/s13567-024-01282-1.

## Introduction

Pyroptosis is a form of lytic programmed cell death dependend on inflammatory caspases such as Caspase-1, -4, -5, and -11, which are activated by inflammasome in response to infection or danger signals [1, 2]. Pyroptosis is characterized by extensive membrane blebbing followed by ballooning of the membrane and eventual loss of membrane integrity [3]. Pyroptosis can defend against intracellular infection by eliminating the compromised cell, thereby removing the protective niche of the pathogen, and releasing cellular contents to elicit an immune response [4]. However, excessive pyroptosis can release numerous intracellular molecules such as DAMP, together with a burst in pro-inflammatory cytokines, resulting in a highly inflammatory response and tissue damage [5]. Cytosolic LPS-mediated pyroptosis was found to be the main driver for sepsis and septic shock [6].

Gasdermin D (GSDMD), as an effector of inflammasome-triggered pyroptosis, is a member of the conserved gasdermin protein family including GSDMA, GSDMB, GSDMC, DFNAS/GSDME, and DFNB59 [7]. GSDMD is cleaved into GSDMD-N-terminal domain (GSDMD NT) and GSDMD-C-terminal domain (GSDMD CT) by inflammatory caspases [8–10]. GSDMD NT oligomerizes into ring-shaped structures to form a large 10–15 nm diameter pore in the plasma membrane, allowing for the passage of IL-1β (4.5 nm in diameter) and IL-18 (5 nm in diameter) [11–14]. GSDMD NT has an affinity for phosphatidylinositol phosphate species, such as PIP1, PIP2, and PIP3, and phosphatidylserine (PS) or cardiolipin, not binding with phosphatidylinositol (PI), phosphatidylcholine (PC), and phosphatidylethanolamine (PE) [12, 13]. Since PIP species and PS are restricted to the inner plasma membrane, GSDMD NT can only form pores from the cytosolic face and does not harm neighboring cells. Furthermore, cardiolipin is a component of bacterial membranes, thus GSDMD NT could kill bacteria, at least in vitro. GSDMD CT could repress pore-forming activity of GSDMD NT by interacting with GSDMD NT in a large surface area [15].

Although the detailed characterization of GSDMD-mediated pyroptosis and the interaction between GSDMD and pathogen in human and rodent systems, our understanding of GSDMD-mediated pyroptosis in bovine is limited. Therefore, this study aims to investigate the characterization of bovine GSDMD in the execution of pyroptosis, including the cleavage site by bovine Caspase-1, critical fragments, or amino acids for pyroptotic activity.

## Materials and methods

### Antibody and reagent

Mouse anti-Flag (AF519) was purchased from Beyotime Biotechnology. Mouse anti-Myc-Tag (#2276), Rabbit anti-GFP (#2956) and Rabbit anti-Gasdermin D (L60, #93,709) antibodies were obtained from Cell Signaling Technology. Mouse anti-GAPDH (AC033) was purchased from Abclonal Technology. Goat-anti-Mouse and Goat-anti-Rabbit antibodies were purchased from Jackson ImmunoResearch.

### Cell cultures and transfection

HEK293T and MBDK cells were obtained from ATCC. HEK293T and MBDK cells were grown in Dulbecco’s modified Eagles’s medium (DMEM) basic supplemented with 10% fetal bovine serum (FBS). Cells were grown at 37 °C in a 5% CO_2_ incubator. Transient transfection of HEK293T cells was performed using JetPRIME (Polyplus Transfection).

### Plasmid construction

Complementary DNA (cDNA) for bovine GSDMD (NM_001359976.1) was amplified from reverse-transcribed cDNA of bovine macrophages. The fragments bGSDMD with or without GFP tag were cloned into p3xFlag-CMV-7.1 or p3xFlag-CMV-14 vectors. The substitution mutants and truncated mutants of bGSDMD were cloned into p3xFlag-CMV-7.1 vector. cDNAs for bovine Caspase-1 and Caspase-3 were synthesized by Tsingke Biotechnolog and cloned into pCMV-Myc vector.

### Cytotoxicity assay

A CytoTox 96 Non-Radioactive Cytotoxicity assay (G1780, Promega) was used to measure cell death. HEK 293 T cells were seeded at a density of 1 × 10^5^ per well in 48-well plates overnight. The cells were then transfected plasmids as indicated for 24 h. To investigate the pyroptosis induced BVDV infection, MDBK cells were seeded at a density of 2.5 × 10^5^ per well in 24-well plates overnight. Subsequently, the cells were infected with BVDV at MOI 1 for the indicated time. Following either transfection or infection, the supernatant of the cells was collected for detection of LDH release.

### Microscopy imaging of cell death

To examine cell death morphology, HEK293T cells were seeded at 1 × 10^5^ per well in 24-well plates overnight for static image capture or in coverslips for fluorescence imaging. Following transfection with the specified plasmids for 24 h, static bright field images of pyroptotic cells were captured using a Nikon ECLIPSE Ts2. To investigate the location of N-GSDMD, cells were incubated with Dil (a red fluorescent probe for cell membrane, Beyotime Biotechnology, C1991S) and DAPI (Beyotime Biotechnology, C1006) for 10 min. Subsequently, cells were rinsed twice with PBS to remove unbound probe. Images were acquired using a confocal laser-scanning microscope (Olympus). The acquired images were processed with Image J software. All image data shown are representative of at least three randomly selected fields.

### Western blot and immunoprecipitation

HEK293T or MDBK cells were lysed using Cell lysis buffer supplemented with a protease inhibitor cocktail (Biomark). The lasytes were centrifuged at 12 000 × *g* for 10 min, and the supernatant was obtained and then denatured in 4 × SDS sample buffer. The protein samples were separated by 12% SDS-PAGE gels and transferred onto PVDF membrane. Membranes were incubated with antibodies and developed using ECL substrate (Bio-Rad). For immunoprecipitations, lysates were incubated overnight at 4 °C with anti-FLAG Beads (Sigma Aldrich). The beads were washed five times with lysis buffer and the immunoprecipitated were eluted with SDS loading buffer and subjected to Western blot assay.

### Statistical analysis

All assays were performed at least three times independently. Data are presented as mean ± SD and analyzed using two-tailed Student’s *t*-test or one-way ANOVA by Prism software (GraphPad). The differences were considered significant when *P* < 0.05 (*), *P* < 0.01 (**), *P* < 0.001 (***).

## Results

### Bovine Caspase-1 cleaves bovine GSDMD to induce pyroptosis

**Figure 1:**
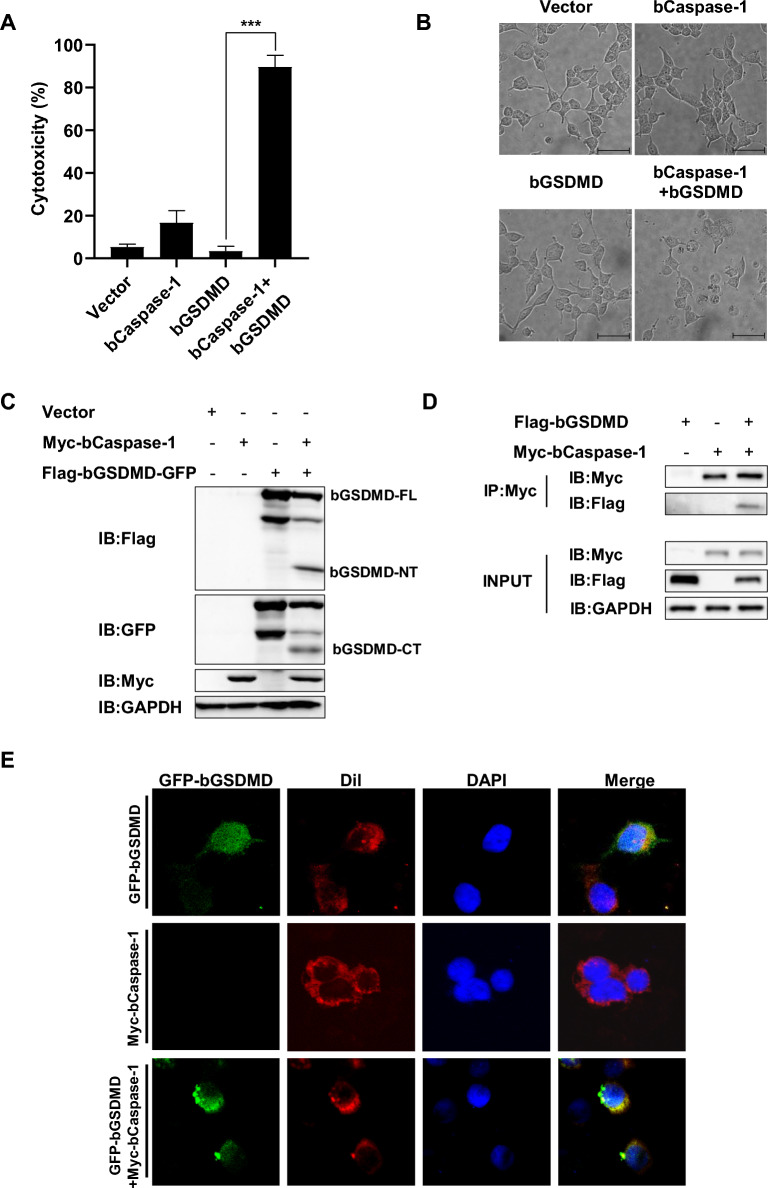
**bCaspase-1 is responsible for bGSDMD cleavage.** HEK293T cells were co-transfected with plasmids encoding bCaspase-1 and bGSDMD for 24 h. **A** The supernatants were collected and analyzed for LDH release levels. **B** Microscopic images of the indicated HEK293T cells. Scale bar, 50 μm. **C** Cell lysates were analyzed with anti-Flag, anti-GFP, anti-Myc, and anti-GAPDH by Western blot. **D** Cell lysates were immunoprecipitated with anti-Myc antibody and then analyzed with anti-Flag and anti-Myc antibodies by Western blot. FL: full length; NT: N-terminal fragment; CT: C-terminal fragment. E Cells were incubated with Dil (a red fluorescent probe for cell membrane) and DAPI for 10 min. The fluorescent signals were observed with confocal immunofluorescence microscopy. * *P* < 0.05, ** *P* < 0.01, *** *P* < 0.001

We employed BVDV as a representative bovine pathogen to investigate pyroptosis and bovine GSDMD (bGSDMD) cleavage in MDBK cells. The results showed that BVDV induced pyroptosis at 24 h and 48 h post-infection (Additional file 1A), while bGSDMD cleavages were detected as early as 6 h post-infection in BDBK cells (Additional file 1B). To investigate the role of bGSDMD in the induction of pyroptosis, HEK293T cells were co-transfected with plasmids expressing bovine Caspase-1 (bCaspase-1) and bGSDMD, respectively. The results, as depicted in Figure 1A, revealed that HEK293T cells co-expressing bCaspase-1 and bGSDMD exhibited a higher release of LDH into the supernatant. Additionally, the co-expression of bCaspase-1 and bGSDMD in HEK293T cells led to extensive cell death characterized by the typical pyroptosis morphology, which includes cell swelling, membrane rupture, and cytoplasmic leakage (Figure 1B). To investigate the cleavage of bGSDMD by bCaspase-1, we constructed a plasmid expressing bGSDMD with a Flag tag at its N-terminus and a GFP tag at its C-terminus. As illustrated in Figure 1C, bCaspase-1 cleaved bGSDMD, resulting in the generation of an N-terminal fragment with a Flag tag (approximately 35 kDa, plus a 2.9 kDa Flag tag) and a C-terminal fragment with a GFP tag (approximately 50 kDa, plus a 26.9 kDa GFP tag). Additionally, we observed an interaction between bCaspase-1 and bGSDMD (Figure 1D). Futhermore, the microscopic examination revealed the presence of N-GSDMD with a GFP tag localized in the cellular membrane (Figure 1E). These findings provide evidence that bGSDMD is cleaved by bCaspase-1, leading to the induction of pyroptosis.

### bCaspase-1 cleaves bGSDMD at amino acid residue D277

**Figure 2:**
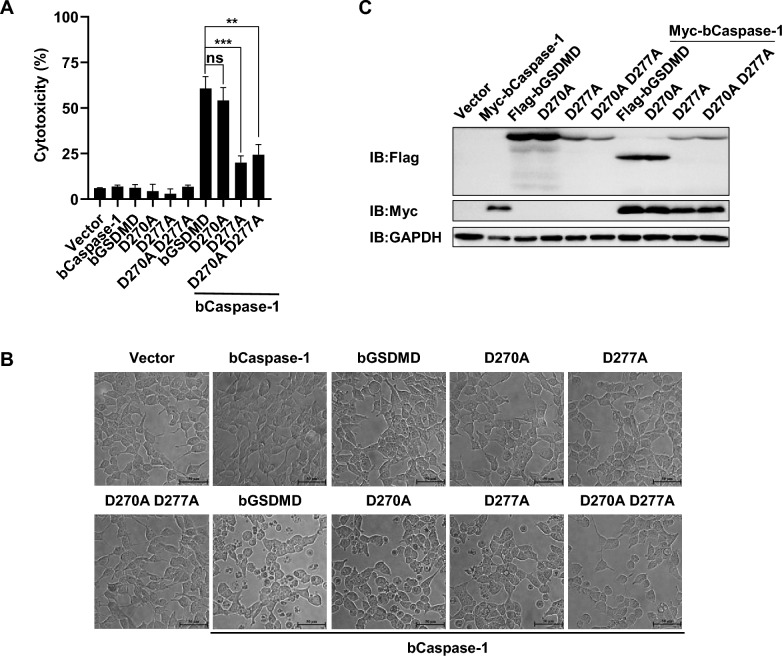
**Residue D277 of bGSDMD is the cleavage site for bCaspase-1.** HEK293T cells were individually transfected with indicated plasmids for 24 h. **A** The supernatants were collected and analyzed for LDH release levels. **B** Microscopic images of the indicated HEK293T cells. Scale bar, 50 μm. **C** Cell lysates were analyzed with anti-Flag, anti-Myc, and anti-GAPDH by Western blot. * *P* < 0.05, ** *P* < 0.01, *** *P* < 0.001

Based on the predicted cleavage site preference of Caspase-1 (XXXD motif) and the sizes of the cleaved fragments of bGSDMD, we performed site-directed mutagenesis to introduce alanine substitutions at the predicted cleavage sites D270 and D277 (Additional file 2), generating single- or double-site mutated forms of bGSDMD. In HEK293T cells, both the wild-type bGSDMD and the D270A mutant induced significant cell death with clear pyroptosis morphology, as depicted in Figures 2A and B. In contrast, the D277A mutant only caused a slight induction of cell death (Figures 2A and B). Furthermore, the D277A mutant resisted cleavage by bCaspase-1 (Figure 2C). The ratio of pyroptosis observed in cells transfected with D270A D277A double mutant was comparable to that of cells transfected with the D277A mutant. These findings indicate that the amino acid residue D277 is the only inflammatory-Caspase cleavage site present in bovine GSDMD.

### Amino acid residues T238 and F239 are key sites for bGSDMD to pyroptosis

**Figure 3:**
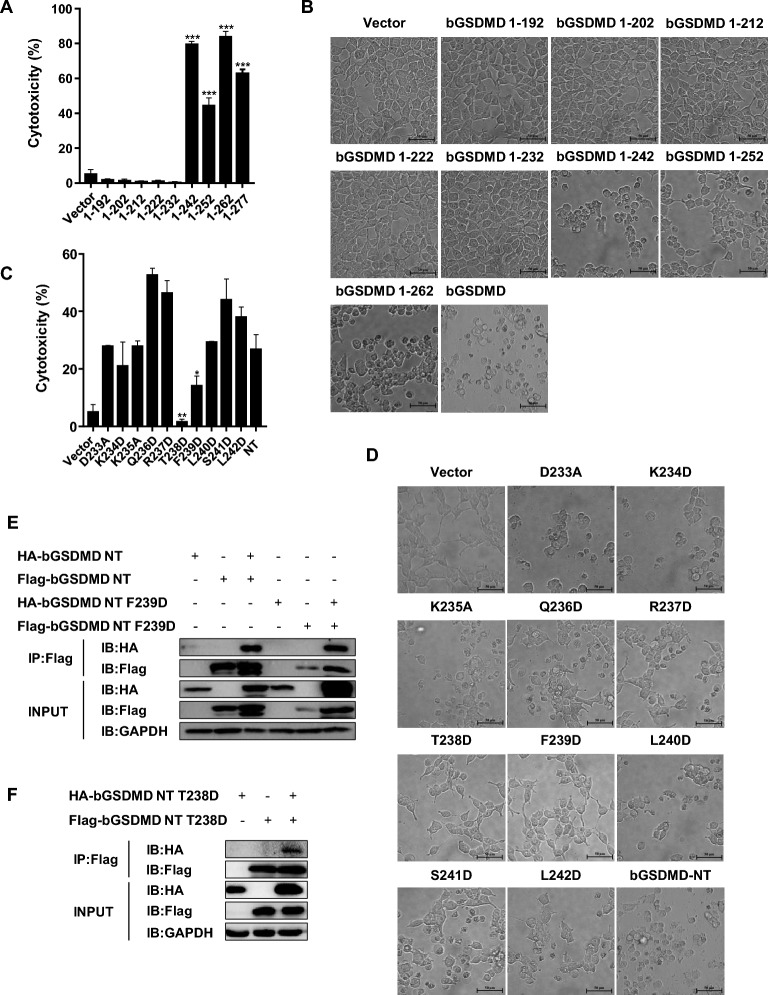
**T238 and F239 are the key sites for bGSDMD NT to induce pyroptosis.** HEK293T cells were individually transfected with indicated plasmids for 24 h. **A** and **C** The supernatants were collected and analyzed for LDH release levels. **B** and **D** Microscopic images of the indicated HEK293T cells. Scale bar, 50 μm. **E** and **F** Cell lysates were immunoprecipitated with anti-Flag antibody and then analyzed with anti-Flag and anti-HA antibodies by Western blot. * *P* < 0.05, ** *P* < 0.01, *** *P* < 0.001

EV71 3C protein cleaved human GSDMD at residue Gln 193 to preclude GSDMD activation, whereas PEDV N protein cleaved porcine GSDMD at residue Gln 193 to inhibit GSDMD activation, indicating that the key fragment to maintain GSDMD NT function is between Gln193 and Asp 275 (Asp279 in porcine GSDMD) [16]. To investigate whether bGSDMD has the same characteristic as human GSDMD and porcine GSDMD, we constructed nine bGSDMD truncated mutants expressing amino acids 1-192, 1-202, 1-212, 1-222, 1-232, 1-242, 1-252, 1-262, and 1-277. These mutants were individually transfected into HEK 293 T. As shown in Figure 3A, HEK 293 T cells expressing amino acids 1-242,1-252,1-262, and 1-272 exhibited a high rate of cell death, whereas those expressing 1-192, 1-202, 1-212, 1-222, and 1-232 did not. The same results were observed in cell morphological features (Figure 3B) and PI staining (Additional file 3A), indicating that the key activity sites located between amino acids 232-242. Next, we constructed the single-site mutants of bGSDMD NT in which amino acid residues between 232 and 242 were replaced by Alanine or Aspartate. The results showed that the T238D and F239D mutants attenuate pyroptosis-inducing activity of bGSDMD NT in HEK293T cells (Figures 3C and D, Additional file 3B). Moreover, we observed T238D and F239D mutants still produced comparable interaction of bGSDMD NT with Flag tag or HA tag compared with wild type (Figures 3E and F), indicating that lower cell death caused by T238D and F239D mutants were not associated with bGSDMD NT self-oligomerization. Collectively, these data suggested that Amino acid residues T238 and F239 are essential sites for bGSDMD NT to induce pyroptosis.

### Functional analyses of bGSDMD autoinhibition

**Figure 4:**
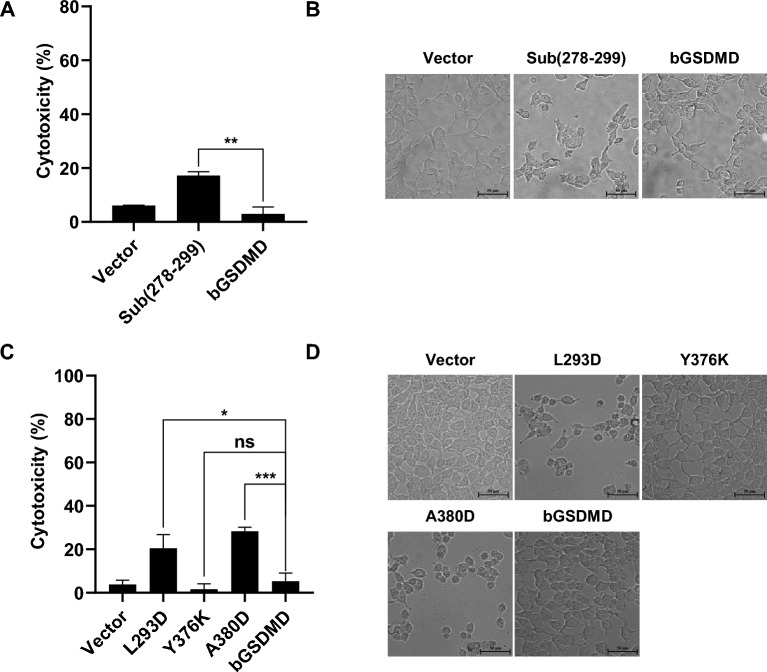
**The loop aa 278–299, L293 and A380 are essential to maintain the autoinhibition of bGSDMD.** HEK293T cells were individually transfected with indicated plasmids for 24 h. **A** and **C** The supernatants were collected and analyzed for LDH release levels. **B** and **D** Microscopic images of the indicated HEK293T cells. Scale bar, 50 μm. * *P* < 0.05, ** *P* < 0.01, *** *P* < 0.001

While GSDMD is intact, GSDMD C-terminal domain (bGSDMD CT) could autoinhibit pyroptosis-inducing activity of bGSDMD NT. The flexible loop (aa 276-296) in human GSDMD inserts into the GSDMD NT pocket, stabilizing the overall structure of GSDMD [15]. Based on the multiple-sequence alignment of GSDMD from human, mouse, porcine, and bovine, we hypothesize that the fragment between aa 278 and aa 299 in bGSDMD might have the same function. We constructed the mutant of bGSDMD in which the fragment (aa278-299) was substituted by a short linker sequence, GSGGGS, without changing the protein quaternary structure. Indeed, the mutant induced more cell death than bGSDMD FL in HEK293T cells (Figures 4A and B). Furthermore, we also investigate amino acids L293, Y376, and A380 in bGSDMD homologous to L290, Y373, and A377 in hGSDMD which have been reported as the key sites in bGSDMD CT autoinhibition. As shown in Figures 4C and D, the results indicated that bGSDMD mutants L293D and A380D rather than Y376 exhibited spontaneous pyroptosis-inducing activity. Collectively, the findings suggested that the loop aa 278-299, L293, and A380 are essential to maintain the stability and autoinhibition of GSDMD.

### bCaspase-3 cleaves bGSDMD without inducing cell death

**Figure 5:**
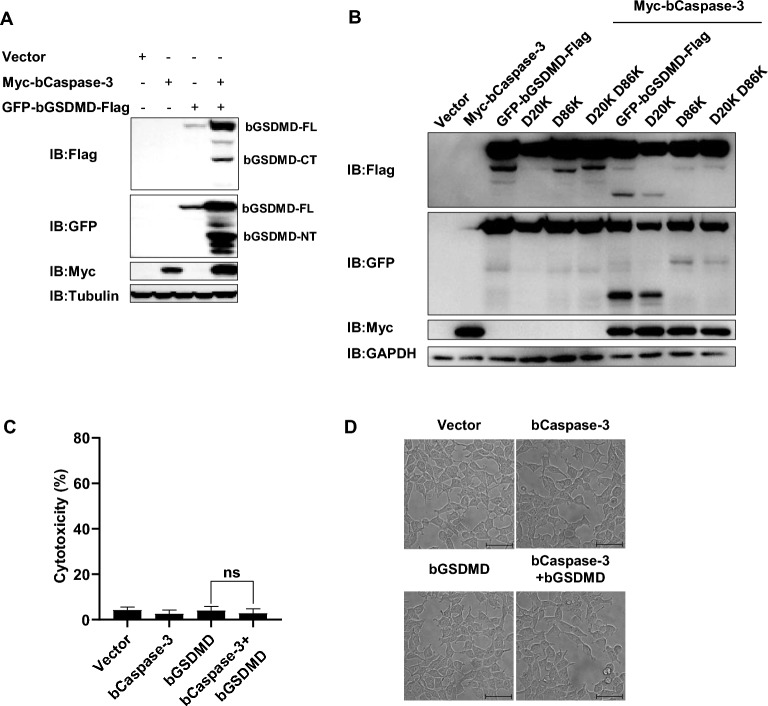
**bCaspase-3 cleaved bGSDMD at residue D86.** HEK293T cells were individually transfected with indicated plasmids for 24 h. **A** and **B** Cell lysates were analyzed with anti-Flag, anti-GFP, and anti-GAPDH by Western blot. **C** The supernatants were collected and analyzed for LDH release levels. **D** Microscopic images of the indicated HEK293T cells. Scale bar, 50 μm. * *P* < 0.05, ** *P* < 0.01, *** *P* < 0.001

It has been reported that bCaspase-3 has capable of cleaving bGSDMD at residue Asp87 during apoptosis. To investigate whether bCaspase-3 has the same function as cleaving bGSDMD, we transfected the plasmid expressing bCaspase-3 together with that expressing bGSDMD with a GFP tag at its N-terminus and a Flag tag at its C-terminus into HEK293T cells. As shown in Figure 5A, bCaspase-3 indeed cleaved bGSDMD into two fragments: the smaller-size N-terminal domain (approximately 37 kDa, plus with 26.9 kDa GFP tag) and the bigger-size C-terminal domain (approximately 46 kDa, plus with 2.9 kDa Flag tag). Based on the Caspase-3 cleavage motif (DXXD), we substituted Asp20 and Asp86 with Alanine. D86A, rather than D20A, was resistant to the cleavage by bCaspase-3 (Figure 5B). However, the cleavage of bGSDMD by bCaspase-3 did not induce more cell death (Figures 5C and D). Thus, the findings indicated that bCaspase-3 cleaves bGSDMD at residue Asp86 without inducing cell death.

## Discussion

Various stimuli or pathogens induce pyroptosis in bovine cells [17–19], however, the precise mechanisms underlying bovine GSDMD-mediated pyroptosis remain unclear. In this study, we show that bovine GSDMD as a substrate for bovine Caspase-1 is essential for pyroptosis. We further elucidate the mechanism of the cleavage site, critical fragment, and key amino acids involved in bGSDMD-mediated pyroptosis. Thus, our research findings provide a detailed elucidation of the mechanism by which GSDMD is involved in pyroptosis, laying the foundation for future research for pyroptosis-related diseases in bovine species.

Prior to our work, human/murine/porcine GSDMD had been demonstrated as a direct substrate of Caspase-1/4/5/11 and serves as the key mediator for pyroptosis [8, 10, 20]. However, the specific amino acid sequence and molecular characterization of bovine GSDMD have remained unexplored. To investigate the molecular mechanism of bGSDMD-mediated pyroptosis, we first confirmed that bCaspase-1 cleaves bovine GSDMD at D277, generating the active peptide bGSDMD-p30, which induces pyroptosis. Furthermore, previous studies revealed that C38/C39 and C191/C192 (human/murine) mutations impaired hGSDMD-p30/mGSDMD-p30 oligomerization [14, 21]. Our results showed that mutation of pGSDMD-p30 T238D or F239D failed to induce LDH release indicating that these two sites are key sites for GSDMD to active pyroptosis. Further investigations are required to determine if GSDMD inhibitors (NSA and NSC) inhibit bGSDMD-p30 oligomerization directly or use alternative mechanisms to suppress bGSDMD-p30-induced pyroptosis. In addition, like other Caspases, Caspase-3 cleaves multiple substrates, including hGSDME [22] and hGSDMD [23]. It has been observed that active caspase-3 inhibits pyroptosis by cleaving hGSDMD at D87 [23]. Consistent with this finding, our study demonstrated that bCaspase-3 cleaves bGSDMD at D86 but does not induce cell death. Therefore, it is reasonable to hypothesize that bCaspase-3 may employ a similar mechanism to suppress pyroptosis in bovine cells.

In conclusion, our study demonstrates that bGSDMD is cleaved by bCaspase-1 at the D277 residue, leading to the initiation of pyroptosis. The specific amino acid residues T238 and F239 are crucial for the pyroptotic activity of bovine GSDMD. Additionally, our investigation into the autoinhibition mechanism of bovine GSDMD sheds light on the regulatory processes involved in pyroptosis in bovine systems.

### Supplementary Information

**Additional file 1: BVDV infection induces GSDMD-mediated pyroptosis.** MDBK cells were infected with BVDV at MOI 1 for 6 h, 24 h and 48 h. A The supernatants were collected and analyzed for LDH release levels. B Cell lysates were analyzed with anti-bGSDMD by Western blot.

**Additional file 2: Alignment of GSDMD amino acid sequences.**

**Additional file 3: T238 and F239 are the key sites for bGSDMD NT to induce pyroptosis.** HEK293T cells were individually transfected with indicated plasmids for 24 h and subsequently dyed with PI for 15 min. The fluorescent signals were observed with confocal immunofluorescence microscopy.

## Data Availability

The data supporting the conclusions of this article are included within the article. Additional data used and/or analysed during the current study are available from the corresponding author upon reasonable request.
