# Supplementary figures and images for: Molecular mechanism of bovine Gasdermin D-mediated pyroptosis
Source: Vet Res. 2024 Feb 27;55:26. doi: 10.1186/s13567-024-01282-1 (PMC10900668; doi:10.1186/s13567-024-01282-1)

**A**

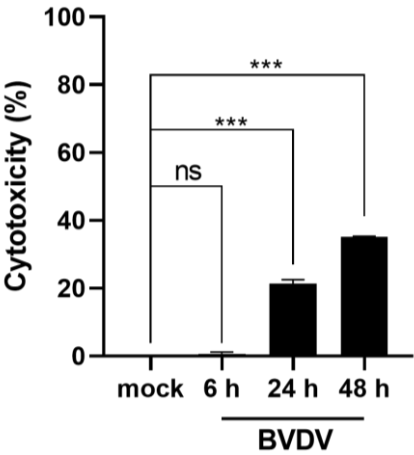

**B**

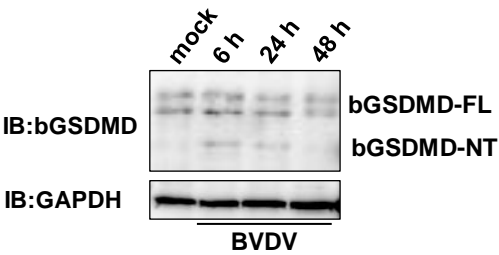

Supplement: Supplementary file 1 — Additional file 1: BVDV infection induces GSDMD-mediated pyroptosis. MDBK cells were infected with BVDV at MOI 1 for 6 h, 24 h and 48 h. A The supernatants were collected and analyzed for LDH release levels. B Cell lysates were analyzed with anti-bGSDMD by Western blot. [file 13567_2024_1282_MOESM1_ESM.pdf]

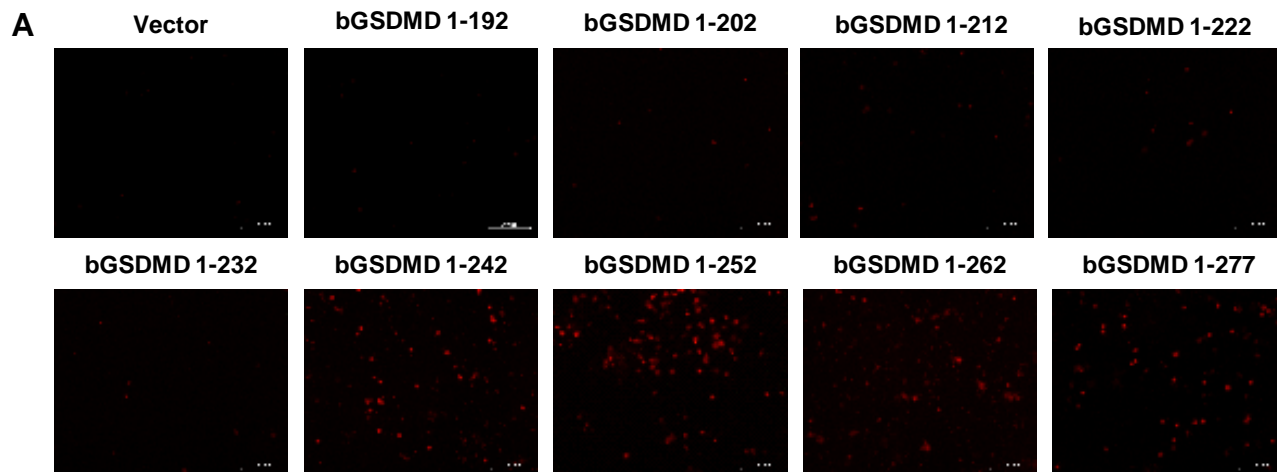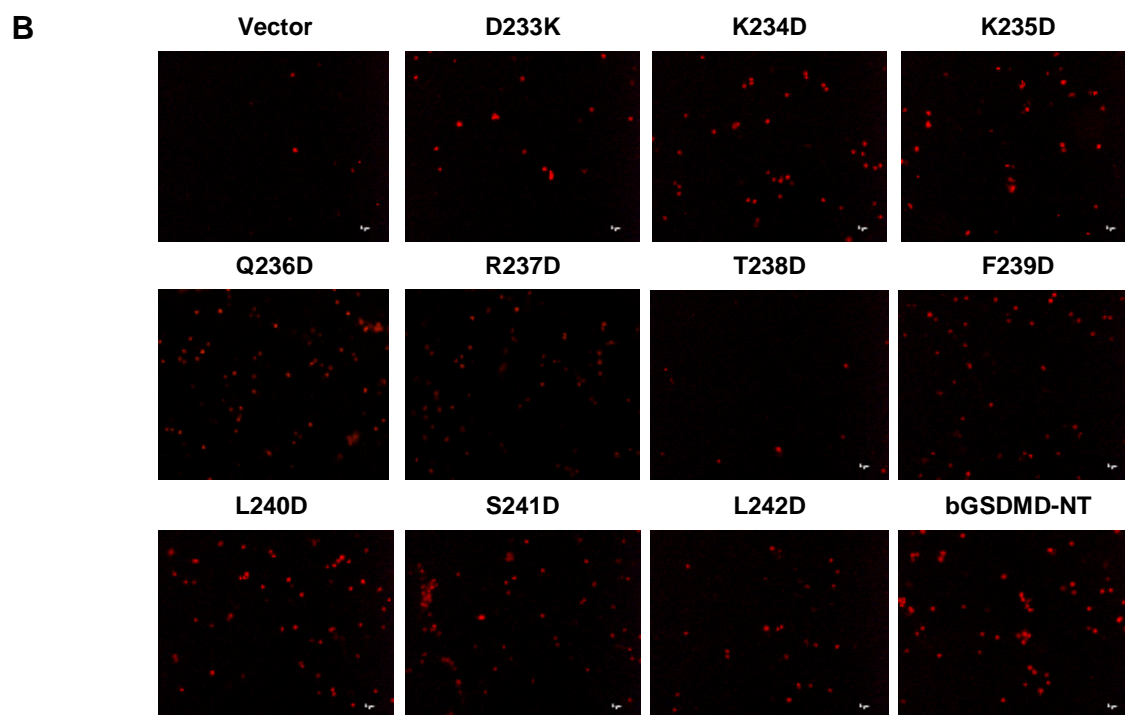

Supplement: Supplementary file 3 — Additional file 3: T238 and F239 are the key sites for bGSDMD NT to induce pyroptosis. HEK293T cells were individually transfected with indicated plasmids for 24 h and subsequently dyed with PI for 15 min. The fluorescent signals were observed with confocal immunofluorescence microscopy. [file 13567_2024_1282_MOESM3_ESM.pdf]
