# Supplementary material for: Molecular mechanism of bovine Gasdermin D-mediated pyroptosis
Source: Vet Res. 2024 Feb 27;55:26. doi: 10.1186/s13567-024-01282-1 (PMC10900668; doi:10.1186/s13567-024-01282-1)
Supplement: Supplementary file 2 — Additional file 2: Alignment of GSDMD amino acid sequences. [file 13567_2024_1282_MOESM2_ESM.pdf]

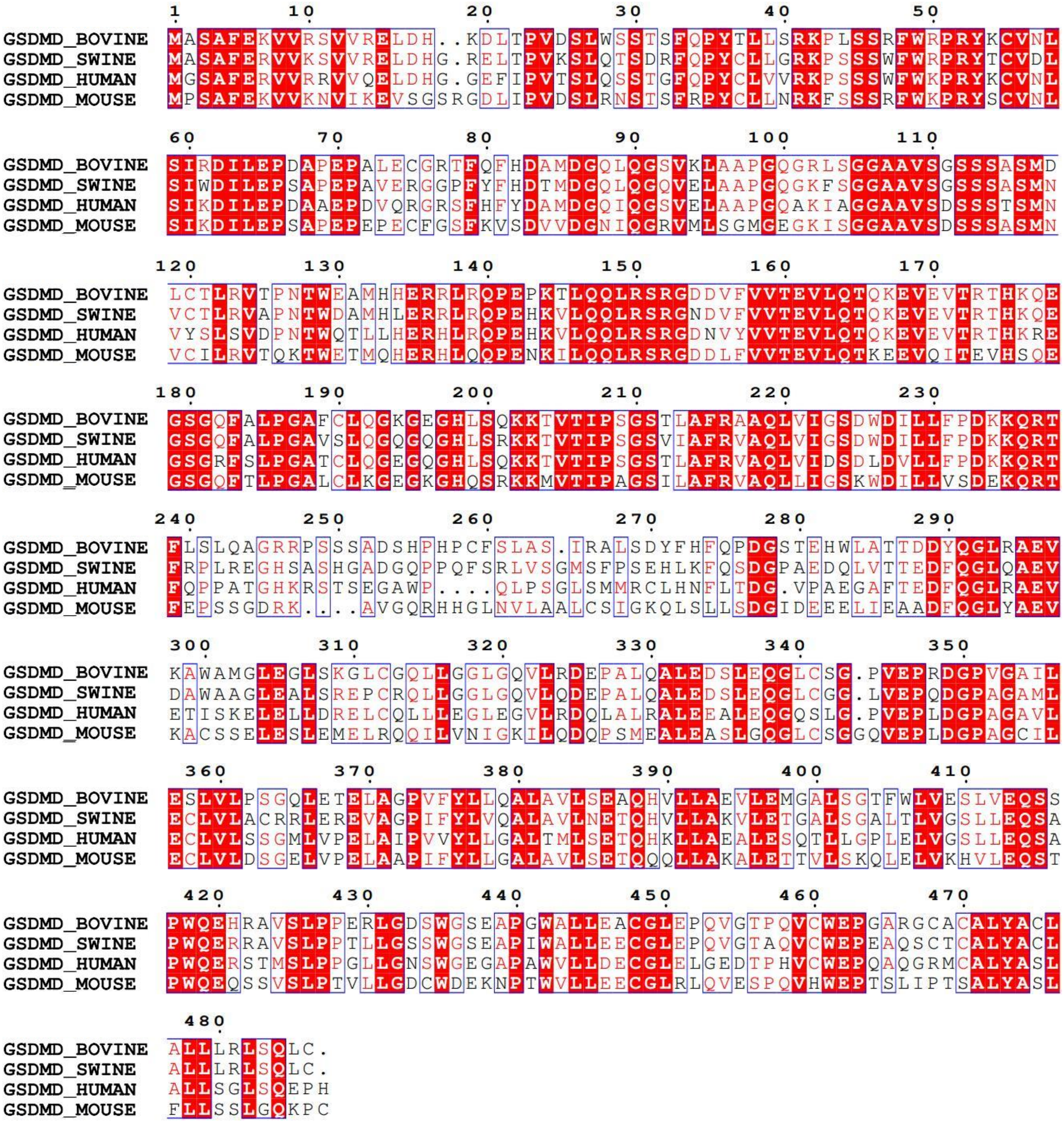

1 10 20 30 40 50
GSDMD_BOVINE MASAFEKVVRSVVRELDH..KDLTPVDSLWSSTSFQPYTLLSRKPLSSRFWRPRYKCVNL
GSDMD_SWINE MASAFERVVKSVVRELDHG.RELTPVKSLQTSDRFQPYCLLGRKPSSSWFWRPRYTCVDL
GSDMD_HUMAN MGSAFERVVRRVVQELDHG.GEFIPVTSLQSSTGFQPYCLVVRKPSSSWFWKPRYKCVNL
GSDMD_MOUSE MPSAFEKVVKNVIKEVSGSRGDLIPVDSLRNSTSFRPYCLLNRKFSSSRFWKPRYSCVNL
60 70 80 90 100 110
GSDMD_BOVINE SIRDILEPDAPEPALECGRTFQFHDAMDGQLQGSVKLAAPGQGRLSGGAAVSGSSSASMD
GSDMD_SWINE SIWDILEPSAPEPAVERGGPFYFHDTMDGQLQGQVELAAPGQGKFSGGAAVSGSSSASMN
GSDMD_HUMAN SIKDILEPDAAEPDVQRGRSFHFYDAMDGQIQGSVELAAPGQAKIAGGAAVSDSSSTSMN
GSDMD_MOUSE SIKDILEPSAPEPEPECFGSFKVSDVVDGNIQGRVMLSGMGEGKISGGAAVSDSSSASMN
120 130 140 150 160 170
GSDMD_BOVINE LCTLRVTPNTWEAMHHERRLRQPEPKTLQQLRSRGDDVFVVTEVLQTQKEVEVTRTHKQE
GSDMD_SWINE VCTLRVAPNTWDAMHLERRLRQPEHKVLQQLRSRGNDVFVVTEVLQTQKEVEVTRTHKQE
GSDMD_HUMAN VYSLSVDPNTWQTLLHERHLRQPEHKVLQQLRSRGDNVYVVTEVLQTQKEVEVTRTHKRE
GSDMD_MOUSE VCILRVTQKTWETMQHERHLQQPENKILQQLRSRGDDLFVVTEVLQTKEEVQITEVHSQE
180 190 200 210 220 230
GSDMD_BOVINE GSGQFALPGAFCLQGKGEGHLSQKKTVTIPSGSTLAFRAAQLVIGSDWDILLFPDKKQRT
GSDMD_SWINE GSGQFALPGAVSLQGQGQGHLSRKKTVTIPSGSVIAFRVAQLVIGSDWDILLFPDKKQRT
GSDMD_HUMAN GSGRFSLPGATCLQGEGQGHLSQKKTVTIPSGSTLAFRVAQLVIDSDLDVLLFPDKKQRT
GSDMD_MOUSE GSGQFTLPGALCLKGEGKGHQSRKKMVTIPAGSILAFRVAQLLIGSKWDILLVSDEKQRT
240 250 260 270 280 290
GSDMD_BOVINE FLSLQAGRRPSSSADSHPHPCFSLAS.IRALSDYFHFQPDGSTEHWLATTDDYQGLRAEV
GSDMD_SWINE FRPLREGHSASHGADGQPPQFSRLVSGMSFPSEHLKFQSDGPAEDQLVTTEDFQGLQAEV
GSDMD_HUMAN FQPPATGHKRSTSEGAWP....QLPSGLSMMRCLHNFLTDG.VPAEGAFTEDFQGLRAEV
GSDMD_MOUSE FEPSSGDRK....AVGQRHHGLNVLAALCSIGKQLSLLSDGIDEEELIEAADFQGLYAEV
300 310 320 330 340 350
GSDMD_BOVINE KAWAMGLEGLSKGLCGQLLGGLGQVLRDEPALQALEDSLEQGLCSG.PVEPRDGPVGAIL
GSDMD_SWINE DAWAAGLEALSREPCRQLLGGLGQVLQDEPALQALEDSLEQGLCGG.LVEPQDGPAGAML
GSDMD_HUMAN ETISKELELLDRELCQLLLEGLEGVLRDQLALRALEEALEQGQSLG.PVEPLDGPAGAVL
GSDMD_MOUSE KACSSELESLEMELRQQILVNIGKILQDQPSMEALEASLGQGLCSGGQVEPLDGPAGCIL
360 370 380 390 400 410
GSDMD_BOVINE ESLVLPSGQLETELAGPVFYLLQALAVLSEAQHVLLAEVLEMGALSGTFWLVESLVEQSS
GSDMD_SWINE ECLVLACRRLEREVAGPIFYLVQALAVLNETQHVLLAKVLETGALSGALTLVGSLLEQSA
GSDMD_HUMAN ECLVLSSGMLVPELAIPVVYLLGALTMLSETQHKLLAEALESQTLLGPLELVGSLLEQSA
GSDMD_MOUSE ECLVLDSGELVPELAAPIFYLLGALAVLSETQQQLLAKALETTVLSKQLELVKHVLEQST
420 430 440 450 460 470
GSDMD_BOVINE PWQEHRAVSLPPERLGDSWGSEAPGWALLEACGLEPQVGTPQVCWEPGARGCACALYACL
GSDMD_SWINE PWQERRAVSLPPTLLGSSWGSEAPIWALLEECGLEPQVGTAQVCWEPEAQSCTCALYACL
GSDMD_HUMAN PWQERSTMSLPPGLLGNSWGEGAPAWVLLDECGLELGEDTPHVCWEPQAQGRMCALYASL
GSDMD_MOUSE PWQEQSSVSLPTVLLGDCWDEKNPTWVLLEECGLRLQVESPQVHWEPTSLIPTSALYASL
480
GSDMD_BOVINE ALLLRLSQLC.
GSDMD_SWINE ALLLRLSQLC.
GSDMD_HUMAN ALLSGLSQEPH
GSDMD_MOUSE FLLSSLGQKPC
